# Acknowledgment to the Reviewers of *Polymers* in 2022

**DOI:** 10.3390/polym15030519

**Published:** 2023-01-19

**Authors:** Polymers Editorial Office

**Affiliations:** MDPI AG, St. Alban-Anlage 66, 4052 Basel, Switzerland

High-quality academic publishing is built on rigorous peer review. *Polymers* was able to uphold its high standards for published papers due to the outstanding efforts of our reviewers. Thanks to the efforts of our reviewers in 2022, the median time to first decision was 13 days and the median time to publication was 33 days. Regardless of whether the articles they examined were ultimately published, the editors would like to express their appreciation and thank the following reviewers for the time and dedication that they have shown *Polymers*:
A. S. M. Monjur Al HossainMałgorzata Kabsch-KorbutowiczAbbas HaddadiMałgorzata Kapelko-ŻeberskaAbbas KhoshnoodMałgorzata Nattich-RakAbdalrhman MiladMalgorzata UlewiczAbdel Basit Al-OdayniMalgorzata ZiebaAbdelaziz M. AboraiaMałgorzata Zienkiewicz-StrzałkaAbdellatif BoukirMalinee SriariyanunAbdelmajid TimoumiMallarapu Gopi KrishnaAbderrahim SamaoualiMalo RosemeierAbdolmajid AlipourManabu ItoAbdorreza Mohammadi NafchiManabu KuroboshiAbdul Qadeer DayoManawwer AlamAbdul QayyumMandakhbayar Nandin-ErdeneAbdul ShaikManfred KrautAbdul Wahab JatoiMani GajendiranAbdullah Al-MamunMani SivakumarAbdulnaser M. AlshoaibiManjunath Patel Gowdru ChandrashekarappaAbdulrahman Al-MalkiManmohan Dass GoelAbdulrahman Al-WarthanManoharan RamamoorthyAbhilash EdacherianManoli M. ZubiturAbhimanyu ThakurManuel Alejandro Fernández RuizAbhinaw KumarMaosud Soroush BathaeiAbhishek DasMaqsood MalikAbimbola E. OluwalanaMarc L. PuseyAbir Said AbdelnabyMarcella BalordiAbobakr Al-SakkafMarcello CasaAbu Zafar Al-MunsurMarcelo AssisAchmad SolikhinMarcia MeierAdam GlinkaMarcin BasiagaAdam MoyseowiczMarcin KoniorczykAdam T. SuttonMarcin MaździarzAdamek GrzegorzMarcin WłochAdarsh K. GuptaMarco EstiAdelaide MirandaMarco FrasconiAdelino V. LopesMarco MorrealeAdem OzcelikMarcos GutiérrezAdeolu Adesoji AdediranMarcus CiceroneAdi SikinMarek FlorkowskiAdina MiclausMarek MackoAditya S. YerramilliMarek PiątkowskiAdnan KhanMarek PokornyAdonilson Dos Reis FreitasMarek UrbanskiAdrian BenitezMargaret GradovaAdrian IoanaMargarita A. KhimichAdrian KnapczykMargarita S. RubinaAdriana BalanMari De MeijerAdriana M. CalvoMaria Angelica MiglinoAdriano A. MendesMaría Arregui GambúsAfonso AzevedoMaria Asun CanteraAfzal Husain KhanMaria BalasoiuAgnė KairytėMaria ChountoulesiAgnieszka Jagiełło-GruszfeldMaria Cristina RighettiAgnieszka Kijo-KleczkowskaMaria D. Rikkou-KalourkotiAgnieszka KrólickaMaria DemeterAgnieszka Skalska-KamińskaMaria Erminia GenoveseAgnieszka WojciechowskaMaria Giacinta PaoloneAgostina OreficeMaria Helena FernandesAgustin EtxeberriaMaria Helena FigueiralAhmad AdewunmiMaría Isabel Rodríguez LópezAhmad Adlie ShamsuriMaría Jose IglesiasAhmad Hussaini JagabaMaria Julia AltubeAhmad Ramazani SaadatabadiMária KováčováAhmed BakryMaria Laura ForestiAhmed BelaadiMaría Laura SalumAhmed FatimiMaria LavlinskayaAhmed Fouzi TarchounMaria LeżańskaAhmed G. El-DeenMaría Luisa González-MartínAhmed HassanainMaría Luisa López-Castejón Ahmed Kadhim HusseinMaría Luisa MoyáAhmed M. YoussefMaría Margarita Suárez NavarroAhmed NafisMaria MarudovaAihua HeMaria NowackaAikaterini Ioannis VavourakiMaria OttAimin ChangMaria Paola LudaAishwarya MenonMaria PomoniAixiang DingMaria Rosaria AcocellaAjay D. PingaleMaria StancaAjinkya DeshmukhMaria StrianeseAjinkya KawaleMarian CosteaAjmir KhanMariana Altenhofen Da SilvaAkbar IdhayadhullaMariana SpineiAkel KanaanMarianne PrévôtAkhil KhajuriaMaricica StoicaAkihito HashidzumeMariia NesterkinaAkikazu ShinyaMarija IvanovicAkiomi UshidaMarija M. BabićAkram HijaziMarija M. VuksanovićAkshay IyerMarija MojicevicAkter HosenMarijana PonjavicAlaa Yassin Al-AhmadMarilia Mattar De Amoedo Campos VeloAlain GuinaultMarina BasagliaAlbert JohnerMarina G. HolyavkaAlbert ManeroMarina I. KozhukhovaAlberto BaldelliMarina KrjuchkovaAlberto GregoriMarina MatosAlcineia OliveiraMarina PaolucciAleck H. AlexopoulosMarina ShavelkinaAlejandra Baldi SevillaMario E. FloresAlejandro Heredia BarberoMario GrassiAlejandro J. AlvarezMariusz DąbrowskiAlejandro J. MüllerMariusz FabijańskiAlejandro Téllez-JuradoMariusz KepczynskiAlejandro VegaMariusz TichoniukAleksandar MiltenovicMariya V. EdelevaAleksander P. SafronovMark A. NannyAleksandr KazachenkoMark B. FramptonAleksandr KozhevnikovMark MoloneyAleksandr M. KulkovMark WilsonAleksandr PoliakovMarkel VinogradovAleksandr S. PozdnyakovMarko BartolacAleksandra BukhovetsMarko MihajlovicAleksandra GrzelakowskaMarkos PetousisAleksandra Janošević LežaićMarkus LahikainenAleksandra Smejda KrzewickaMarlene Lariza Andrade GuelAleksandrs VolpertsMarli Luiza TebaldiAleksei Vorob'evMarta Fiedot-TobołaAleksey D. DrozdovMarta Gonzalez-AlvarezAlena VimmrováMarta MarszałekAlena Yu. YaryshevaMarta Miotke-WasilczykAlessandra LongoMarta WiśniewskaAlessandro MarradiMarta ZacconeAlessandro PiovanoMartha E. PoisotAlessio MezziMartin BednarikAlex Elías-ZúñigaMartin DankoAlex Yong Kwang TanMartin HubbeAlexander BittnerMartin RosentrittAlexander BungeMartin TaylorAlexander ChenMartin TichýAlexander G. KolpakovMartina ContranAlexander GagarinMartina KlucakovaAlexander KapustinMary Lyn C. LimAlexander MarinMaryam Azizi LalabadiAlexander MolnarMaryam Ghiyasiyan-AraniAlexander NaumkinMaryam HasanpourAlexander PestovMary-Carmel KearneyAlexander PogrebnjakMarzena JamrógiewiczAlexander S. AbyzovMarzena Jędrzejczak-KrzepkowskaAlexander TarasovMarzena Włodarczyk-StasiakAlexander TsouknidasMarzio RancanAlexandra GyongyosiMasafumi YanoAlexandra KhlyustovaMasahiko YamaguchiAlexandra ShakunMasahiro AkiyamaAlexandre PoulhazanMasakazu MorimotoAlexandre VetcherMasaki OmiyaAlexandre ZakharovMasanori YamadaAlexandru Isaic-ManiuMasaomi IkedaAlexey SivokhinMasaru MukaiAlfonso García-MárquezMasataka KuboAlfredo Téllez ValenciaMasayoshi HiguchiAlfredo Vázquez-OvandoMasoud Nazarian-SamaniAli Abdulkhaleq AlwahibMasoud Vesali-NasehAli BahariMasoumeh GhalkhaniAli Farokhi NejadMasoumeh OzmaianAli KhavaninMassimiliano Matteo PellegriniAli MoshkrizMassimo DuranteAli RazaMassimo GazzanoAli Reza RezvaniMassimo LazzariAli Shaan Manzoor GhummanMassimo MarielloAli Shaygan Nia Mateescu CarmenAli TaghizadehMatej MičušíkAliakbar HassanpouryouzbandMateja KertAlina Crina MureşanMateusz IwańskiAlina DutaMateusz SzczygiełdaAlina MateiMatheus PolettoAlireza HadiMatjaž KristlAlireza MoradkhaniMatjaž PavličAlison ScottMatthew DonaduAlperen DegirmenciMatthew J. RegulskiÁlvaro GonzálezMatthew WadgeAlvaro YogiMatthias J. RoggendorfAlwar RamaniMatthias KroegerAmaia Lejarazu-LarrañagaMatthias LexowAmal NassarMattia ConteAmanda PacholakMauricio A. Sarabia-VallejosAmany RagabMauricio Alves Da Motta SobrinhoAmar BallabhMauro BallicchiaAmarinder Singh SinghMauro MalvèAmaury Pozos-GuillénMaxim KhramchenkovAmber SolangiMaxim MishnevAmbra GuarnaccioMayank PatelAmbrish SinghMd. Nizam UddinAmin KhajehdezfulyMd. Nuralam HossainAminat UzdenovaMd. Reazuddin ReponAmir Abbas Shayegani AkmalMd. Rejvi KaysirAmir Ershad-LangroudiMd. RizwanullahAmir MofidiMd. Sahid HassanAmir RostamiMd. Salatul Islam MozumderAmir ZadaMd. Shahruk Nur-A-TomalAmirjalal JalaliMedhi RiddhimanAmit AlexanderMegha ChitranshiAmit JaiswalMehdi DerradjiAmit Kumar VermaMehdi HatamiAmit SharmaMehmet IsikAmitava MoulickMehmet KarahanAmmar A. MelaibariMehmet KodalAmol V. PansareMehrab NodehiAmrindra PalMeimei LaiAmrita KunduMeitian XiaoAmritlal MandalMelgardt De VilliersAna AlvesMelinda SzekelyAna Beatriz Morales CepedaMenake PiyasenaAna D. Popović-BijelićMeng PuAna De SousaMengjie ShouAna Gonzalez NoyaMengmeng LiuAna Lúcia HorovistizMengmeng ZhangAna Maria GalanMengtao SunAna Maria RosuMengying YangAna Paula ChungMengyuan WangAna TomićMeram AbdelrahmanAnanto AlhasyimiMercedes FernándezAnastasia D. PournaraMershen GovenderAnastassia MashentsevaMert TunaliogluAnastassia N. RissanouMessaouda BoumaazaAnatoli PopovMeta MahendradattaAnatoly E. ChalykhMetin OrnekAnda BarkaneMiće JakićAnders AxelssonMichael AdabreAndrás LengyelMichael ArulAndré LehmannMichael BertocchiAndré Luiz MissioMichael CiesielskiAndré MeloMichael CromerAndrea BoghiMichael PissasAndrea ButeraMichael SchulzAndrea LabouriauMichał DrożdżekAndrea Maria GuarinoMichał ŁobaczAndrea MartinelliMichał RogalaAndreas EndruweitMichal RymsAndreas LambertzMichel Picanço OliveiraAndreea L. Chibac-ScutaruMichele FerrariAndrei OkhokhoninMichele Placido Antonio GattoAndreia RibeiroMichele TerzanoAndreja R. LeskovacMieke BuntinxAndrejs KovalovsMiguel A. García-SánchezAndres Sanz GarciaMiguel Ángel Hidalgo SalazarAndrew B. CrollMiguel MachadoAndrew NaylorMiha SteinbücherAndrey A. ZhirnovMihaela Cristina BaicanAndrey ApelfeldMihaela FloriAndrey MilchevMihai A. GîrțuAndrey NagdalianMihai EftimieAndrey SarikovMihaiela IliescuAndrey ShcherbakovMihail MihaylovAndrey YakovlevMike BambachAndrii KoveriaMikhael BechelanyAndrii MaryninMikhail A. ArtemovAndrij PichMikhail A. MatskoAndryushin Konstantin PetrovichMikhail KarushevAndrzej ChwojnowskiMikhail PetrovAndrzej Jarosław PanasMikhail PorozhnyyAndrzej KulczyckiMikhail SkripkinAndrzej LeskiMikihito TakenakaAneesh KoyappayilMikko KanervaAneta WieczorekMiklós KubinyiAngela MarottaMikołaj BernasowskiAngelo Earvin ChoiMilad SheydaeiAngelo TagliettiMilan Babu PoudelAnh HuynhMilan StehlíkAnida Maria BabtanMilena Álvarez-ViñasAnil Kumar SirohaMiloš PánekAnila IqbalMiloslav MilichovskýAnimesh DebnathMilton Cesar Costa CamposAniruddha MondalMilutin M. MilosavljevićAnisa KaurMina NamvariAnita KwaśniewskaMine SeçkinAnjan BediMing JiangAnjanapura V. RaghuMing LiAnke KlingnerMing QuAnkit GangradeMingchao ZhangAnkit VoraMinghao LiuAnn Sofi JönssonMingkang SunAnna KazachenkoMingsheng ChenAnna Marin-GozalboMingtao ChenAnna ShipovskayaMingze SunAnna StrzelewiczMingzhi ZhangAnna SygudaMinhgung TruongAnna SzymczykMinjee KangAnna TrusekMir Saman SafaviAnna Zimoch-KorzyckaMiran MerharAnna ŻywickaMirela RantaAnne LaPointeMirella Romanelli Vicente BertoloAnouar El MagriMiriam F. ScelzaAnthimos AnastasiadisMiriam ScotiAnthimos S. AnastasiadisMirjana JovicicAnton BlencoweMiroslav MensikAnton PashkevichMiroslava ŤavodováAnton S. TkachenkoMiroslaw GalazkaAnton ZubrikMirosław GiurgAntonella GrigolettoMisu MoscoviciAntonella MacagnanoMizera KamilaAntonija TadinMizhi XuAntonino FamulariMladen MarkovićAntonino PolimenoMo YangAntónio AlmeidaMochamad AsrofiAntonio AngeloModestas ŽiliusAntonio BartolomeoMohab El-HakimAntónio DelgadoMohamad Fakhrul Ridhwan SamsudinAntonio Dominguez-AlfaroMohamad Hafiz MamatAntonio PérezMohamed Ahmed TahaAntonio TrovatoMohamed Bechir Ben HamidaAntonio Valverde-GonzálezMohamed DamejAntonios G. StamopoulosMohamed ElsafiAntonios N. PapadopoulosMohamed EltaherAntreas KantarosMohamed HasaninAntxon Martínez De IlarduyaMohamed HewidyAnukorn PhuruangratMohamed M. F. DarwishAnup JoshiMohamed Mahmoud El-Sayed NasefAnup PandithMohamed SalaheldeenAnuraag BoddupalliMohamed SharafeldinAnurag DubeyMohamed Thariq SultanAnurag PisupatiMohammad A. HasnatAnurag SharmaMohammad Abdul WahabAnusorn SeubsaiMohammad AkramiAnvay PatilMohammad FasihiApostolos KorlosMohammad H. AlyamiApostolos VagiasMohammad Ilyas KhanApri Heri IswantoMohammad Islam Araceli FloresMohammad Javed AnsariAram BahmaniMohammad Kazem Hassanzadeh-AghdamAravind PedarlaMohammad Mirazul IslamArbind PrasadMohammad Mizanur RahmanAree ChoodumMohammad MohsenzadehArijit SenguptaMohammad Qasim ShaikhArit DasMohammad Reza Naimi-JamalArkadiusz GendekMohammad TaufikArman Moini JazaniMohammad ThaljiArmando Roman-FloresMohammad YosofvandArmida Sánchez-EscalanteMohammad Z. KabirArnoldo LopezMohammad Zia AlaviArseni V. UshakovMohammadreza ElahifardArtem MarchenkovMohammadreza RezaeigolestaniArtem PushkarevMohammed AbbasArthur MerkelMohammed Abdulhadi SarhanArtur AndrearczykMohammed AwadArtur ValenteMohammed BourhiaArun ButreddyMohammed GhazwaniArun K. SinghMohammed HawashArun Kumar PatraMohammed MajdoubArun PatilMohammed SayedArun Prabhu RameshbabuMohammed Seddik MeddahArũnas RamanavičiusMohammednoor AltarawnehArunkumar PalaniappanMohandoss SonaimuthuAsad UllahMohd Abul KalamAseem ChawlaMohd Bijarimi Mat PiahAshang LaivaMohd BuraidahAshish AgrawalMohd Firdaus OmarAshok Kumar BalaramanMohd Mahadi HalimAshraf El-BindaryMohd Nasrun Mohd NawiAshutosh KumarMohd Salahuddin Mohd BasriAsli YilmazMohd Shaiful SajabAsmaa M. FahimMohd Shukry Abdul MajidAsmaa SelimMohsen YazdanianAssunta CampanileMojtaba Lezgy-NazargahAtanu ChakrabortyMona T. Al-ShemyAtefeh KarimzadeharaniMonica PeriolattoAtefeh ZarepourMonika BožíkováAtiq Ur RehmanMonika Dobrzyńska‐MizeraAtsushi AsanoMonika KovačevićAtsushi KobayashiMonika KuźniaAtsushi MorikawaMonika PooniaAttila GergelyMonika Śmiga-MatuszowiczAtul DhallMoniruddoza AshirAtul SharmaMonita OliviaAudrone SankauskaiteMoreno MarcelliniAugustine Osamor IfelebueguMostafa BaghaniAurelia Cristina NechiforMostafa MabroukAurélien DeniaudMoti Lal RinawaAurore FraixMoud Aref AbbasiAykut ErbaşMourouzis PetrosAylin M. DeliormanliMoustafa M. G. FoudaAyoub MoghadamMoustafa Mahmoud Yousry Mahmoud ZaghloulAyse DundarMousumi De SarkarAzadeh AsefnejadMridul UmeshAzat GabdulkhakovMrinal BhattacharjeeAzeman Nur HidayahMrinal KaushikAzile NqomboloMrinmoy KarmakarAziz AmineMuayad Al-ShaeliB. NagarajaganeshMuchao QuBabar AliMuhammad Akhsin MuflikhunBachir Raho GhalemMuhammad ArifBadawi AnisMuhammad AsifBahador BahramiMuhammad AtiqullahBahareh TanhaeiMuhammad AyyoobBaji ShaikMuhammad Faisal JavedBakhtiyar OrazbayevMuhammad HarrisBaltej Singh RupalMuhammad Hazwan HamzahBang HeMuhammad IjazBangshang ZhuMuhammad Imtiaz RashidBankole I. OladapoMuhammad IqbalBanruo HuangMuhammad KashifBao JiaMuhammad MujtabaBarbara GierobaMuhammad Muzamil KhanBarbara HajdukMuhammad Nadeem ZafarBarbara JadachMuhammad QaiserBarbara KozubMuhammad QamarBàrbara Micó VicentMuhammad RizwanBarbara MulloyMuhammad SaqibBarbara TomadoniMuhammad ShahidBartłomiej KostMuhammad Tariq QamarBarun Kumar BarmanMuhammad Usman HameedBasanta Kumar BiswalMuhammad Wahab AmjadBasheer ThazeemMuhammad ZahidBasudeb MondalMuhammad ZamanBeata BorakMuhammet CeylanBeata Butruk-RaszejaMujtahid KaavessinaBeata DudziecMulham AlfatamaBeata KorchowiecMunder BilemaBeata StaniszMuralimohan CheepuBeata Świeczko-ŻurekMuralithran KuttyBehnam AkhoundiMurat DemiralBéla PályiMuriel AmielhBelviso Benny DaniloMurugesan RajaBénédicte LepoittevinMusa AdamuBenjamin BatchelorMushriq AbidBenlin HuMustafa Erden YildizdagBenoit LoppinetMustafa FawzyBernardeta DębskaMustafa Sezar DincerBernardo Gonçalves AlmeidaMu'tasime Abdel-JaberBernardo RibeiroN. Rajesh Jesudoss HynesBerwin Singh Swami VethaNa ZhouBeyza Ünalan DeğirmenciNaba KalitaBharati NeelamrajuNabarun SahaBhausaheb TawadeNadeem BaigBianca Maria TihăuanNadeem RazaBill DavidsNadia AkramBimlesh LochabNadia Mahmoudi KhatirBin HongNafei HeBin LiuNafisa GullBin ZhangNagaraj NagaprasadBing DuNagarajan SrinivasanBingxi YanNaimeh BahriBinling ChenNajm M. Al-HosinyBinoy MaitiNajmedin AziziBiplob BrmanNamasivayam AravindBishnubrata PatraNamchul ChoBlanca Lorenzo VeigaNamil KimBlaž LikozarNamkyung LeeBogdan Marian TofanicaNan LiBogdan Viorel NeamțuNanda Gopal SahooBogumiła KumanekNandhakumar RajuBor-Jiunn WenNanditha AnandakrishnanBoubié GuelNaofumi NagaBo-Yeon LeeNaraindas BheelBoyko G. TsyntsarskiNaresh KumarBożena KarolewiczNarmina O. BalayevaBranislav MarkovićNashrul Fazli Mohd NasirBranka AndričićNashwa HagagyBrenda G. MolinaNatali Murri AnnalisaBrian MeldeNatalia Andrea TarazonaBright Singh SeeniNatalia Evgenievna KruchininaBritani BlackstoneNatalia GladkikhBrittan ScalesNatalia KarpukhinaBuddhi PushpawelaNatalia PozdnyakovaBudimir MijovićNataša SamardžićBulat AkhmadeevNathan CowiesonBulat SaifutdinovNathan GallantBurak AlakentNathdanai HarnkarnsujaritByeong Hee KimNaveed NidaByoung Seong JeonNaveen KumarCamil LanceaNawroz TahirCamilo Zamora-LedezmaNazia JamilCancan JiangNectarios VidakisCândida MalçaNeetu TalrejaCândida TomázNeli KosevaCarl AnthonyNelson E. CoatesCarl Martin BellNelson S. BellCarlos A. T. TolozaNemanja GavrilovCarlos Andrés Palacio GómezNemanja P. TrišovićCarlos Doñate BuendiaNewcomb RobertCarlos DrummondNgoc A. NguyenCarlos F. O. GraeffNicholas F. MatererCarlos MarcuelloNicolas DelpouveCarlos MoyaNicolas MuzzioCarlos NegroNicolas RollandCarlos Santiuste RomeroNicolas SöhlingCarlos Vinicios OpeltNicole Y. MorganCarme GüellNidhin DivakaranCarmen Alice TeacǎNihal SarierCarmen MitaNikhitha JosephCarol BarryNikita YushinCarola Esposito CorcioneNikola BregovićCarola MuranoNikola GligorijevićCarolina CaicedoNikola MaravićCarsten DoscheNikola ŠpanićCatalin AmzaNikola ZdolšekCécile NouvelNikolaos ChalmpesCelestino Garcia-GomezNikolaos NazirisCelina BernalNikolaos SilikasCerne BorutNikolay A. PyataevCesar SaldiasNikolay Melik-NubarovCeyhun AksoyluNikolett Kállai-SzabóCezary KraśkiewiczNilesh ChoudharyChadrasekhar LokaNima MohamadianChae-Ho YimNima ShahkaramipourChak-Shing KwanNimal Thattaruparambil RaveendranChaminda BandaraNina PastorChan I. ChungNing LiChanchal Kumar KunduNing WangChanchira JubsilpNino GrizzutiChandra MadasuNirmal MazumderChang GuoNisarg ModiChang LiuNisha ChoudharyChanghai ChenNishant RanjanChanghao LiuNishesh SharmaChangqing FangNitesh MittalChangyue ChiangNitin K. ChaudhariChan-Woo ParkNitin Krishnamurthy HansogeChaobo HuangNiyaz MahmoodiChaolin LiuNizar MeksiChaoyaug LiaoNobuhiro KawatsukiCharles PaulNoelia MachadoChayanaphat ChokradjaroenNoor Saeed Khan KhattakChe Ku AbdullahNoorina Hidayu JamilChenglong LianNorbert Krisztián KovácsChengmin JiangNorbert NemesChengming TangNorberto FeitoChenguo WangNorhana JusohChengwei LinNorihiro InagakiChengyang MoNorma Sofía Torres PabónChenhung HuangNorman S. AllenChenyu KaoNourouddin SharifiChenyu LiNowsheen GoonooChetan TambeNuman YanarCheyu LinNur Alim BahmidChiache WuNuttapol TanadchangsaengChiajung ChoOana Emilia ConstantinChiara RinoldiOana Viorela NistorChien WernOgnen Pop-GeorgievskiChihchiang HongOgnjen PerišićChihchien ChuOkikiola AgbabiakaChihhong LinOlatokunbo M. OfuyatanChinghsuan LinOleg KorobeinichevChinmay SatamOleg ManaenkovChinwen ChenOleg SazonovChi-Ping LiOleg StolbovChirag GodiyaOleg V. GolubevChiranjeevi KorupalliOleg V. GradovChitien ChenOlga DymshitsChitrangada Das MukhopadhyayOlga L. VoroninaChongyu ZhuOlga LebedevaChris GreetOlga SamoilovaChristian ChapaOlga SerenkoChristian GorzelannyOlga TrhlíkováChristian HaritoOlga UrbanekChristian HöhneOliver WeicholdChristian SchmitzOllie Yiru YuChristian TantardiniOmair MohiuddinChristian ZafiuOmar Garcia-ValdezChristiane A. HelmÖmer CivalekChristos A. GogosOmer SaleemChu Yee KhorOmid NabinejadChuan Li LeeOndrej JurcekChuang WangOnur YilmazChun Hung ChuOsamu KamiyaChunhuan ZhangOscar De LucioChunhui XiangOskar J. BeraChunju LiOskars PlatnieksChunming YangOsman ErkmenCinthia Maia PederneirasOsvaldo Agustín LambriCíntia Helena Coury SaraceniOswaldo BaffaClara López-IglesiasOvidijus ŠernasClastinrusselraj I. SathishOvidiu S. StoicanClaudia DessiOxana V. KharissovaClaudia DettoriOzgul GokClaudia SayerOzlem Ipek Kalaoglu-AltanClaudia SergiPablo BlancoClaudio CaprariPablo Lopez-AlbarranClaudio ImparatoPablo Vila LameiroClemens K. WeissPablo Zapico GarciaClement MugemanaPanagiota KarahaliouCodruta VarodiPanagiotis DallasColette LacabannePanagiotis SpyridisConcha TojoPanagiotis ZoidisCongling ShiPantelis KourosConstantin GeorgescuPantu Kumar RoyConstantin NechitaPanuwat JoykladCorneliu HamciucPaola Serena GinestraCosimo LigorioPaolo CarrubbaCostas A. AnagnostopoulosPapović SnežanaCostas TzoganakisParamanandam ThomasCraig M. ClemonsParas N. YadavCristian DeacParaskevi PapadopoulouCristian PeptuParisa MojaverCristina Antonela BanciuParthena Maria KosmidouCristina Gabriela GrigoraşPaschalina TerzopoulouCristina M. S. G. BaptistaPasha TabatabaiCristina PelinPathapalli Venkateshwar ReddyCristina SchreinerPatricia Losada-PerezCristina Tristão AndradePatrícia Manarte-MonteiroCsilla PesznyákPatrícia Schmid CalvãoCyrille RichardPatricio Orellana-PalmaCzang-Ho LeePatrick CelieDabin GuoPatrik RohnerDaeIk JangPatrizio SaliceDaiqin ChenPatryk JedrasiakDai-Soo LeePaul D. EwartDaisuke IshiiPaul RöschDaiva MikucionienePaul SottaDalibor ŠatínskýPaul SundaramDamián Reyes-JáquezPaul TophamDamla KeskinPaula RodriguesDan AokiPaula Villanueva-LlauradóDan Mihai ConstantinescuPaulina KasprzykDana AkilbekovaPaulina Latko-DurałekDana PerniuPaulo Díaz-CalderónDaniel ChuchalaPaulo H. S. PiccianiDaniel Fernández-QuirozPaulo Jorge AntunesDaniel GromadzkiPaulo SantosDaniel Heras MurciaPavel ChapalaDaniel IghigeanuPavel KomarovDaniel L. Z. CaetanoPavel PleskunovDaniel Olvera TrejoPavel ProsrDaniel P. UraPavel RehakDaniel VeghPavel S. PostnikovDaniela Herrera-BalandranoPavel V. KomarovDaniela JumancaPavel ZelenikhinDaniela KunecováPavla ČapkováDaniela MilevaPavol FarkašDaniele BattegazzorePawan KumarDaniele ChiriuPaweł BrykDaniele MantionePawel MajewskiDaniele Mirabile GattiaPawel PietaDaniele Ribeiro De AraújoPawel ZylkaDaniil N. BratashovPayal GangulyDanijela ŠuputPedram AzariDanilo Martins Dos SantosPedro CruzDanka Labus ZlatanovicPedro FernandesDanko MiloševićPedro MartinhoDanna TangPedro Ojeda-MayDaojun LiuPedro RoblesDapeng ChenPedro SimõesDaqian GaoPedro Veiga RodriguesDaria PoshinaPei Song CheeDarinka FakinPeilong HouDario LasićPeiman ValipourDariusz PykaPeiran WeiDariusz SykuteraPeisheng LiuDarya RadziukPeiyuan QuDaryn BorgekovPeng BoDave MangindaanPeng WuDavid ArencónPengcheng XieDavid AyrePere CastellDavid BeggPeriyasami GovindasamiDavid BergbreiterPetar PetrovDavid De SmetPete WildeDavid DixonPeter KotešDavide GardiniPeter Ó ConghaileDavide MandelliPeter O'ConnellDavide NocitaPeter R. LaityDavood RahmatabadiPeter ZarrasDavor KovačevićPetko AlovDavoud BalarakPetr KoudelkaDe GongPetr SyselDebin XiaPetre IonitaDebipreeta BhowmikPeyman PourafsharyDebra E. WhiteheadPhilip YuyaDeep KalitaPhilipp RöseDehui WangPhilippe CassagnauDelong HePhilippe DumasDelong LiPia Willberg-KeyriläinenDenis RodriguePiaoran YeDenise BellisarioPierre MertinyDenizhan YavasPietro AusielloDennis FlanaganPietro MazzucaDennis Richard LajeunessePilgun OhDessy RachmawatiPing’an SongDezhong YangPingcheng HsiehDhanasingh Sivalinga VijayanPiotr BoruszewskiDharamdeep JainPiotr M. ZielińskiDharmapura MurthyPiotr PrzybylekDharneedar RavichandranPiotr ZabierowskiDhorali GnanasekaranPiyawanee JariyasakoolrojDhwani KansalPo-Jung HuangDi JiaPolina TyubaevaDian YuanPolona Dobnik DubrovskiDiana ChioibasuPouyan GhabeziDiana CholakovaPowen ChenDiana PopescuPradeep Kumar PandaDiana SerbezeanuPraful R. NairDidier R. LongPrakash BhuyarDieter HohenwarterPramod K. KalambateDiletta AmiPramod Kumar SinghDilhun Keriman Arserim UcarPranut PotiyarajDimas RamboPrasanna RamaniDimitrios BikiarisPrashanth RavishankarDimitris C. TsamatsoulisPrasit ThongbaiDimka I. IvanovaPrathap SomuDina MendonçaPratik KoiralaDina MoussaPraveen SherDinesh BhalothiaPredrag DašićDinesh Kumar SharmaPřemysl PařenicaDinesh PatelPriscilla AmaralDinesh Veeran PonnuveluPriyadarshini JayashreeDineshkumar SengottuveluProbal BasuDingying ZhouProloy Taran DasDipak RanaPrzemysław DopieralskiDipen Kumar RajakPrzemysław GolewskiDirk VanderzandePrzemysław KosobuckiDixit BhalaniPulak BhushanDizaj Solmaz MalekiPunta CarloDjalal TrachePunyaslok RathDmitry BuslovichPurba PurnamaDmitry KiesewetterQasim Shaukat KhanDmitry S. KosyakovQi RaoDo Yeung YoonQian ZhangDobrochna Ginter-KramarczykQiang GuoDoga BilicanQianqian ZhangDomagoj BelicQibin LiDomenico SagnelliQifeng MuDominic WalesQiguang HeDominika JaniszewskaQihan LiuDong GuoQihui WangDong WangQijin ZhangDong-Jun KwonQin XuDonglin ZhaoQing ChenDora Allegra CarboneQing WangDorina Nicolina IsopescuQingchang LiuDorota Ciołczyk-WierzbickaQinghua WangDorota Czarnecka-KomorowskaQingshun DongDorota DziurkaQinqin ZhangDorota PawlusQiucheng LiDorota PuchowiczQiyao HuangDorota Wójcik-PastuszkaQuang Minh NgoDovan ThomQuentin BourgogneDragica SpasojevićQuoc Bao LeDrahomír ČadekR. Bharath VenkateshDuc Dung NguyenRachana SahneyDuc-Thang VoRachel Yerushalmi-RosenDungdinh LuongRachid HsissouDunja Šajn GorjancRadmila Jančić HeinemannDuong NguyenRadoslav SovjákDurai PrabhakaranRadovan DojcilovicDurcilene SilvaRaelene CowieDursun SaraydınRafael CuestaDušan KopeckýRafael DrampyanDushyant BarpagaRafael Vargas-BernalDuško DudićRafał JanuszewskiDyicheng ChenRafał PetrusDylan AnstineRaffaele CiardielloDzun Noraini JimatRaffaella BucciEbenezer AnnanRafiq AhmadEbubekir KayaRahim AbdurEdgars ElstsRahul K. GuptaEdina LempelRahul KumarEdmar Oliveira-FilhoRaj KumarEduardo A. Lopez-MaldonadoRaj Kumar AryaEduardo Festozo VicenteRaj MukherjeeEduardo GuzmánRaja GhoshEduardo N. FrankRaja RamanathanEdward LoukopoulosRajamani DevarajEdward RenRajan KrishnaprasadEdyta KucharskaRajeshkumar SelvarajEdyta MałachowskaRajib BandopadhyayEftekhari AzizRajkishor KumarEftihia BarnesRakesh BhaskarEgor DontsovRakesh RajaboinaEhsan ArshidRakhi TiwariEhsan BehzadfarRam DattEhtasham MustafaRama Sreekanth Pattela SrinivasaEkaterina NaumenkoRamachandran ManickamEkaterina PrikhozhdenkoRamachandran VinayagamEl Hadi EribiaiRamakrishna DadigalaElaheh Dalir AbdolahiniaRamasamy BhoopathiElaheh Kafshdare GoharshadiRamasubbareddy PalemElaine CardosoRamesh RudrapatiElder Alpes De VasconcelosRaminder KaurÉlen Beatriz Acordi Vasques PachecoRamzi Hadj LajimiElena A. SilantyevaRan TaoElena DinteRana Faisal TufailElena GolubevaRando TuvikeneElena López-MayaRanhua XiongElena ManailaRaquel De L. C. GiordanoElena NikolskayaRashid NazirElena PopaRasim AlosmanovElena RomeoRasmina HalisElena TomsikRastislav LagaňaElena V. KudryashovaRavi Kant GuptaElena-Raluca BaciuRavi TutikaElena-Suzana Biriș-DorhoiRawen SmiraniEleonora SocoRaymundo CaseEleonore FröhlichRayssa Ferreira ZanattaElettra PapaRazvan RadulescuElham Hosseini NejadReaz ChowdhuryElham PishavarRebar AbdulwahidEliana CapecchiReda SalamaElisabete Pereira Dos SantosRegina M. IslamovaEliza ChircanRenata Salgado FernandesElnaz EsmizadehRencheng DongElsa Díaz-MontesRenjang WuElumalai SanniyasiRenjie LiuEly ValbuenaRenyuan QinElyas Asadi ShamsabadiRethinam SenthilElżbieta BociągaRewati Raman UjjwalElżbieta Hać-szymańczukReza DarvishiElżbieta K. HorszczarukReza KolahchiElżbieta SzostakReza NorooziEmad Abdul-Hussain YousifReza PeymanfarEman KhalafRhoda Afriyie MensahEmanuel Hernández NúñezRiadh NeffatiEmanuela SperanziniRicardo Hernández-MartínezEmanuele LocatelliRicardo KamikawachiEmanuele RizzoRicardo M. F. FernandesEmerson CoyRicardo SilvaEmi Govorĉin BajsićRiccardo BeltramiEmil KhisamutdinovRiccardo MonterubbianesiEmil SasimowskiRichard D’ArcyEmilia IrzmańskaRichard HaverkampEmily C. DavidsonRichard HrčkaEmmanouil Lazaros PapazoglouRichard PinčákEmmanouil VougioukasRichard PriceEmrah MadenciRichard T. TaylorEn WangRidhwan JumaidinEnfeng DengRika OchiEnrique Castro-CamusRim BourgiEnshirah Da'naRina Dhirajlal KoyaniErdogan MadenciRishi ThakkarErfan OliaeiRobert E. PrzekopEric FujiwaraRobert GuidoinEric NuxollRobert JurczakErick Juarez-ArellanoRobert Kwok-Yiu LiErik AndreassenRobert OpilaErik Steen RedekerRobert SchennachErnesto ReverchonRobert StarostaErol YildirimRoberta D’AurelioErwin WojtczakRoberto Juan Jose WilliamsEsam Abdel-HadyRoberto PastoreEsfandiar PakdelRoberto PetrucciEslam El-SawyRobin HutchinsonEssia HannachiRocco Di GirolamoEtienne DucrotRocío RedónEugen Mircea AnitasRocio Yaneli Aguirre LoredoEulalia GliścińskaRoddel RemyEusebiu Ilarian IoneteRodrigo FrançaEva IvanišováRodrigo ResendeEva María García-FrutosRoger FrétyEvan Wenbo ZhaoRoger HiornsEvando AraújoRoger R. DraheimEvangelos KarvelasRogerio Sousa JuniorEvangelos ManthosRok FinkEvgenia V. SalomatinaRoland SchochEvghenii HareaRoman BoroznjakEwa DłuskaRoman ElashnikovEwa GlowinskaRoman PichugovEwa Olewnik-KruszkowskaRoman PopielarzEzequiel M. Vázquez-LópezRoman V. ChernozemFabiano S. RodembuschRoman V. DeevFabienne PeyrotRomeo CiobanuFabio De MatteisRonald Huarachi OliveraFábio Filipe Fereira GarrudoRong MaFabricio Maestá BezerraRongchun ZhangFabrizio DavìRonghui WuFabrizio OlivitoRoobanvenkatesh ThirumalaiFacundo MatteaRoozbeh Abedini NassabFacundo RuizRosa María LozanoFadis F. MurzakhanovRosalva Mora-EscobedoFahad M. AlmutairiRosario González-MotaFahad UsmanRostislav ApraksinFaheem Arjamend SheikhRoszalina RamliFahmi F. MuhammadsharifRovia KobunFaiz AliRowena BruggeFakhri ShahidiRubén F. González LaredoFalah AbuRubén González-NúñezFaliang LuoRudolf PfaendnerFangcheng LiangRudolf ZentelFania PalanoRuei-Tang ChenFanny Monteil-RiveraRufina AlamoFarhad SharifRui ChenFarhana AbedinRui DaiFaridah SonsudinRui HuangFarzad KermaniRui SunFarzan GholamrezaRui XiaoFarzaneh SabbaghRuimeng ZhangFatih OzRuinian JiangFatma KocakRuiqing ShenFatmanur Tuǧcu-DemirözRuitao WuFawaz AldabbaghRukiya MatsidikFederica BuccinoRuming PanFederico BellaRungtiva P. Poo-ArpornFei FanRunzhou HuangFei LiuRupinder SinghFeifei ZhouRuslan SuleymanovFeipeng YangRüstem KeçiliFeiyu XuRuth M. MuthokaFeng JunRuth-Sarah RoseFeng TangRyo IshiharaFengmei SuRyohei IshigeFerdinand DobešSabapathy ThennarasanFerenc RonkaySabarinathan P. SubramaniyanFerenc Tolvaly RoșcaSabina NilufarFernanda BoniSabornie ChatterjeeFernanda VilarinhoSabyasachi GhoshFernando C. G. MartinhoSachin S. NaikFernando Cepero-MejíasSachin SalunkheFernando ElySadaf GilaniFernando GalembeckSaeed AshtianiFernando J. PereiraSafeer AhmedFethi AbbassiSagar PardeshiFilippo SamperiSahand SarbisheiFioretta AsaroSahar MollazadehFlorent BarbaultSaheli ChakrabortyFlorian OlivierSai Aditya PradeepFlorian SpieckermannSaif UllahFlorina M. BojinSaiful Izwan Abd RazakFranc VrečerSaikat Sinha RayFrancesc EstranySajal SenFrancesca BoggioSajjad MalikFrancesca MiglioriniSakandar RaufFrancesco CiardelliSaket PatelFrancesco Degli InnocentiSakine ShekoohiyanFrancesco Marotti De SciarraSakshum KhannaFrancesco Paolo La MantiaSalah-Eddine StiribaFrancesco PotenzaSalawu SulymanFrancesco TornabeneSaleh Hassanzadeh GharaieFrancis VocansonSalihu IbrahimFrancisco Eroni Paz Dos SantosSalman AliFrancisco Javier López-TenlladoSalvatore IannaceFrancisco Javier Navarro DomínguezSalvatore ScolavinoFrancisco Lopez-LinaresSaman GhahriFranck CamerelSamarendra MajiFrancois MoriniSameer A. AwadFrancois VidalSameh A. AhmedFrank BalzerSamntha MacchiFranscious CummingsSamuele CiceriFranz Josef MaringerSanchita KunduFranz StelzerSanda Liana BaltesFulga TanasăSanda Mihaela PopescuFurong TianSandeep AryaFuyin HsuSandip PalGabriel A. MontañoSandor SolyomGabriel Luna-BarcenasSandra CorreiaGabriel MansourSandra MendozaGabriel Ramos-OrtizSandro DattiloGaël ColominesSandro Jose Ribeiro BonattoGamal MoustafaSang Moon KimGang LiuSangeet AdhikariGang XuSangmo KooGaofeng ZhengSang-Young YeoGarry KerchSanika SuvarnapathakiGary S. GrestSantanu BasakGautham JeppuSantanu MukherjeeGaweł SołowskiSantanu PanjaGeetanjali DeokarSantidan BiswasGeetanjali ShuklaSantosh PeddiGennadiy A. KostinSapana JadounGennaro Salvatore PonticelliSaqib FarooqGeorgia KastrinakiSara BaldassariGeorgios BokiasSara BernardiGeorgios E. ArnaoutakisSara Garzon-HernandezGeorgios ParaskevopoulosSara HedayatiGeorgios SkordarisSara HernandezGera SoniaSara PedronGerasimenko Alexander YuryevichSarah GlaßGerhard SinnSarai Agustin-SalazarGhanshyam TejaniSaranshu SinglaGhassan AlmasabhaSarfarazi VahabGheorghe NagitSascha HeitkamGheorghe PaltaneaSascha RohnGholamhosein MoloudianSathish Kumar PalaniappanGholamhossein LiaghatSatish ShilpiGia Toai TruongSatoshi KodairaGiacomo CanaleSatuluri SrikiranGiacomo CecconeSaulius JuodkazisGibin GeorgeSaurabh MishraGideon RotichSawsan AlmohammedGil GarroteSayan BanerjeeGina AmbrosioSayed SalehGina Delia Roque-TorresSean-Thomas B. LundinGinka K. ExnerSebastian DahleGiovanna IucciSebastian Marian ZahariaGiovanna MurmuraSébastien AlixGiovanni CagnettaSeh-Hoon OhGisele Amaral-LabatSeisuke AtaGiulia MoroSelim GurgenGiulio MalucelliSelin Kinali-DemirciGiuseppe CirilloSelvin P. ThomasGláucia DalfréSenad DizdarGo IgarashiSenka PopovićGökhan AcikSenthil Muthu Kumar ThiagamaniGokhan SerhatSeog-Jin JeonGonzález-Torres MaykelSeonhwa ParkGonzalo Martínez BarreraSerene Sow Mun LockGopinathan JanarthananSerge PerezGoran PalijanSergei KomogortsevGrazia Giuseppina PolitanoSergei TverdokhlebovGreta DonatiSergey I. MoseenkovGrigorios RaptopoulosSergey LarinGrzegorz DomekSergey LyulinGrzegorz JanowskiSergey ShevtsovGrzegorz KowalskiSergey SolomevichGrzegorz MakomaskiSergey Yu. SavinGrzegorz SochaSergio Antonio Marques LimaGrzegorz SzparagaSérgio Frascino Muller De AlmeidaGrzegorz WoroniakSergiusz LulińskiGuanglei WuSertac KarakasGuangling JiaoSeung Koo ParkGuangyong ZengSevakumaran VigneswariGuanhua WangSeverine Le RoyGuanjie HeSevil Savaşkan YilmazGuiance Laura EstévezSevki CesmeciGuido GoracciSeyed Mohammad MazloomiGuilherme CaetanoSeyed Mohsen SerajiGuiliang ChenSezgin ErsoyGuillaume Miquelard-GarnierShadeed GadGuillaume VivesShadrack FosuGuiwei LiShafiq IshakGülay BaysalShafiullah KhanGunasunderi RajuShah AbbasGunawan Setia PrihandanaShahid AdeelGünter LorenzShahid Ali KhanGunther BrunklausShahid IqbalGuo Dong GohShahood Uz ZamanGuodong DengShahram AjoriGuoli TuShahriar BalaniGuoxing LuShaik Gouse PeeraGurpreet SinghShaikh A. AliGururaj Kudur JayaprakashShakhawan AlzanganaGustavo GonzálezShalini Jayaraman RukmaniGustavo Henrique NalonShang JiaHabibullah NadeemShanganyane Percy HlangothiHacı SaglamShangli DongHafez JafariShangzhi ChenHafiezal RadziShankar Jaikishan EvaniHai XinShankar KharalHaipeng YuShanshan ChenHaitao CuiShaolong SunHaiyang A. YuSharanabasava V. GanachariHaiyi LiangSharif Montassar AidiHalyna OharShatil ShahriarHamdy F. M. MohamedShayfull Zamree Abd RahimHamed Aghajani DerazkolaShehla MushtaqHamed DabiriSheikh Ahmad Izaddin Sheikh Mohd GhazaliHamed Salimi-KenariShen WangHamid OsmanShenbaga Velu PitchumaniHamid Reza HamediSheng LiHamidreza PirayeshSheng PanHamit AdinSherif MehannyHammad BadarSheyda ShakibaHan HanShi TangHan Joo KimShib Shankar BanerjeeHanan Osman-PonchetShibananda DasHani Nasser AbdelhamidShichang TsengHans-Matti BlenckeShihhuang TungHanyi DuanShijian WuHao GuShikinaka KazuhiroHao MeiShing-Yun ChangHao ShenShin-Ichi KiharaHao YuShin-Ichi YusaHaoran QuShin-Ichiro TohmuraHaoyan WeiShinji MiyataHarekrushna SutarShiuhhwa ShyuHarminder Pal SinghShivanjali SharmaHarnoor Singh SacharShixing WangHaroon Ur RasheedShixue WangHaroutioun AskanianShogo TakamukuHarsh VardhanShoune XiaoHartmut GliemannShoyebmohamad F. ShaikhHasan F. AlesaryShravan MuthukrishnanHassan A. AlshahraniShrestha Sinha RayHassan AlbarqiShreya VemugantiHassan ChamatiShruthi DasappaHassan GhonaimShu WangHatim AlotaibiShu ZhangHazel AssenderShuai QianHe LiangShuai XieHe SunShuang ShiHe ZhaoShuangxi WangHéctor GarcíaShubha NageswaranHefeng ZhangShubhangi KhadtareHelena Oliver-OrtegaShubhankar BhattacharyyaHelena TeteryczShuchuan LiaoHélène GarayShudipto Konika DishariHemant JainShudong LinHemant SinghShujahadeen B. AzizHemanth GudapatiShuko SuzukiHend AbdelhakimShunichiro ItoHeng DaiShunyi JianHengkwong TsaoShuyang YeHenrique Almeida SantanaShyhshin HwangHeraldo Luiz GiachetiSi LiHerbert SixtaSibali DebnathHeriberto Hernández-CocoletziSid Ahmed SlimaneHideaki KatogiSidharth RazdanHideharu MoriSidi ZhuHidekazu KawashimaSilvestre Bongiovanni AbelHideki HayashiSilvia BarbiHidemasa KatsumiSilvia Becerra-BayonaHideyuki SatoSilvia DanteHikaru KoboriSimon Bambang WidjanarkoHiroaki SaiSimon StephanHirohisa TamagawaSimon WangHirosato MonobeSinan EğriHiroshi KoibuchiSintu RongpipiHiroshi YokotaSirilux PoompradubHiroyuki AokiSirinya ChantarakHiroyuki TakeiSiti BaidurahHisaki WatariSiti Hasnah KamarudinHon Huin ChinSiti Noorbaini SarminHong ZhaoSiu Hong Dexter WongHongda LuSivasankar AnnamalaiHongjian ZhouSivoney De SouzaHongli LiSixun ZhengHongxi LuoSiyang YangHoracio VieyraSkibiński SzymonHornyák IstvánSlađana DavidovićHosein NaderpourSlavica MaleticHossein AbbastabarSmrithi PadmakumarHossein EslamiSnežana BabićHossein KabirSnezana Ilic-StojanovicHossein MinoueiSobhi DanielHossein MokhtarianSofya YarusovaHossein NassiraeiSohail Anjum ShahzadHossein Ramezani-DanaSohrab RahimiHrčka RichardSongsong LiHristo P. PenchevSonja Herres-PawlisHuaguang YangSorb YesudhasHuanda ZhengSoslan A. KhubezhovHubert MorańdaSotirios G. StavropoulosHugo FerreiraSotiris PatsiosHui HuangSoumen DasHui ShenSoundaram Jeevarathinam AnanthakrishnanHuifang XuSourav ChatterjeeHuihong LiuSovan BanerjeeHuiling LiSpiro D. AlexandratosHung NguyenSravendra RanaHungyin LinSrećko ManasijevićHusnul Azan TajarudinSreekar Babu MarpuHussein Hamel ZghairSri Kalyana Rama JyosyulaHwa RyuSri MeliaHyang Moo LeeSridhar ChandrasekaranHyeuk Jin HanSriharsha Srinivas SundarramHynek BalcarStanica EnacheIan NichollsStanislau BoguszIbrahim AbdellahStanislav ObručaIbrahim AlshaikhStanislav PatlazhanIbrahim DubdubStanisław KucielIbrahim M. GadalaStanisław MlekoIbrahim M.A. MohamedStanley Udochukwu OfoegbuIbrahim MohamedStavros PoulopoulosIdalina J. DomingosStavros TsantzalisIeva MisiūnaitėStefan KöstlerIfeyinwa I. ObianyoStefan SchröderIgnacio BallesterosStefania PorcuIgnacio JessopStefano AneliIgor E. VyaliyStefano BellucciIgor NovakStefano GiordanoIhab ShigidiSteffen WitzlebenIlgım GöktürkStephan KubowiczIlian HristovStephanie VivodIlias IliopoulosSteven W. RickIllyas Bin Md IsaSubaer JunaediIlsiya M. DavletbaevaSubhendu BhandariIlya KhodovSubing QuImad Shakir AbboodSuchada KongkiatkamonIman YazdiSucharat LimsitthichaikoonImelda Olivas-ArmendárizSuchithra Ashoka SahadevanImre KissSudhakar NarraIn Woo CheongSudipta DasInderbir SinghSue NangIndrek MustSugato HajraInmaculada Concepción Suárez MuñozSuhail KhanIntan Rosalina SuhitoSuhana Binti KotingIoan PeteanSuiping DengIoannis A. AsimakopoulosSukanta BhowmickIoannis KaratasiosSukanta DasIon CalinaSumedha LiyanageIon SavaSu-Mi HurIonuţ Ovidiu TomaSumit KumarIrena IvaniševićSundararajan RamaswamyIrene LingSung Jae JeonIrene Perez-AlfaroSung Woon ChaIrene S. FahimSungmin HanIrene TagliaroSungmin KangIrfan BahiuddinSupaphorn ThumsornIriczalli Cruz-MayaSuping ShenIrina CherunovaSurachai KarnjanakomIrina PopescuSurendra NimeshIrina SulaevaSusana MendesIrina V. SemenovaSusanta Kumar BhuniaIrineu BatistaSushanta SethiIrwin Rose Alencar MenezesSusumu MitsuyamaIsaac Montoya De Los SantosSuwat NananIslam A. KhalilSven KarlovićIsmaeil GhasemiSven RutkowskiIsmael Fuente MerinoSven SaengerlaubIsra AliSvetislav SavovicIsrael GonzálezSvetlana C. GagievaIsrafil KucukSvetlana GrushevskayaIsrar UddinSvetlana KamzolovaIstvan SzaboSvetlana KlementevaIvan FortelnýSvetlana PolivtsevaIvan GajdošSvetlana V. KononovaIvan GiorgioSvetlana Z. RogovinaIván Herrero-DuráSwami Vetha Berwin SinghIvan HevusSwapnali HazarikaIvan IzoninSwee Leong SingIvan KozyatnykSyed Ibrahim Gnani Peer MohamedIvan R. QuevedoSyed MahmoodIvan ShatskyiSyed Muhammad ImranIvan ShepaSyeda Abida EjazIvan V. TerekhovSylva HolešováIván Valdivia-GandurSylvain CaillolIvana AleksićSylwia ŻołądekIveta CabalovaSzabolcs FischerIveta MarkováSzymon DawczynskiIvica ÐuretekSzymon MalinowskiIwona GolonkaSzymon StarzonekIwona KarbownikT. M SridharIwona RykowskaT. Umasankar PatroIzabela BetlejTachita Vlad-BubulacIzabela IrskaTaghreed Khaleefa Mohammed AliIzabela JasińskaTahir MuhmoodIzabela KruszelnicaTahmid Hasan RupamIzabela MiturskaTaira MiyasakaJacek Adam SorokaTakahiko NakaokiJacek LewandowiczTakaki KanbaraJacek LubczakTakashi KajitaniJacek OlenderTakayuki IkeharaJacek PacanaTakumitsu KidaJacek PezdaTakuya HayashiJacek SiódmiakTakuya IsonoJacinto Efrén Ramírez-BribiescaTakuya MaeyamaJacob C. LutterTalal S. AmhadiJadranka MilikićTalat BaranJadwiga SołoduchoTalib M. AlbyatiJaehan JungTamara Fernández-ArévaloJaime Rubio-MartinezTamás TábiJakub MatusiakTamerlan T. MagkoevJamal M. RzaijTanakorn Phoo-ngernkhamJamarosliza JamaluddinTanja ZidaričJames DudleyTapan Kumar PalJames Kit-Hon TsoiTariq AzizJan BobrowiczTariq DarabsehJan CzyzewskiTat Thang NguyenJán IždinskýTatjana M. PuškarJan PaczesnyTatjana ŠoštarićJan SzadkowskiTatjana Volkov-HusovicJan TrejbalTatyana LugovitskayaJan ZidekTatyana P. ShakhtshneiderJana SedlaříkováTawfik KhattabJan-Christoph ZargesTejas BarotJandeep SinghTeng ChiJanis RizhikovsTeodora StaicuJanja TrcekTeofil JesionowskiJanuar Parlaungan SiregarTerence A. ImberyJanusz LubasTeresa AdityaJarosław ChodórTeresa Fina MastropietroJasmine SinhaTeresa RucinskaJasna Simonović RadosavljevićTeresa SibillanoJavier Echávarri OteroTerjék AnitaJavier IllescasTetsuya HoriuchiJavier Zamudio-GarcíaTetsuya TakahashiJay AiraoThais Suzane Milessi EstevesJayalakshmi AyyavooThanh Binh Nguyen ThiJayaraman TheerthagiriThanh Cuong-LeJean Aime MbeyThanigaivelan KanagasabaiJean Christian BernèdeThawatchai PhaechamudJean-Daniel MartyTheodor DollJean-François AgassantTheodoros ManourasJean-Luc PutauxThi Kim Hoang TrinhJean-Marc HaudinThi Minh Phuong NgoJean-Michel NunziThi Tuong Vy PhanJȩdrzej WalkowiakThiago F. Da ConceicaoJeff MaThiago SequinelJeffrey EvertsThierry RibeiroJeffrey G. LundinThomas Fuhrmann-LiekerJelena D. VujancevicThomas M. GradzielJelena PetrovićThomas PatersonJelena ŠkamatThomas SchweizerJennifer HulingThomas SeppererJens SchusterThomas VoglhuberJens WippermannThomas ZöggJeong KimThorsten PretschJeongguk KimThuan Ho-Nguyen-TanJérémy OdentThuat TrinhJerzy ChruścielThuy Quynh Truong HoangJerzy J. LangerTianhao YuJesus CastrelloTiciane Santos ValeraJesus Manuel Fernandez OroTim SchubertJesús MengualTina SabelJesús Puente-CórdovaToan Thanh DaoJeyan MosesTom SunnyJia WeiTomas JandaJiahao ChenTomas JanuševičiusJiahao HuangTomás Madera-SantanaJiahe WuTomáš TrčkaJiahuan JiangTomasz GubicaJiahui ChenTomasz KlekielJiajia YinTomasz KloskowskiJialin MaoTomasz KrawczykJialong ShenTomasz RozwadowskiJian ShengTomasz StrachowskiJiangtao LiuTomasz UchaczJianhua FangTomasz WasilewskiJianlei WangTomica HrenarJianwen WangTomislav SedlarJianxun DingTommaso D'AntinoJianzhu JuTomonaga UenoJianzhuang XiaoTong ShenJiayi YuTongsai JamnongkanJie LiuTongye ShenJigneshkumar PatelTonya AndreevaJihong LiuTooba ShoaibJimena S. GonzalezToshihisa TanakaJin CaoToshiyuki KanakuboJin Chul YangTraian RotariuJin MotoyanagiTung Ping WangJinal PothupitiyaTurgay SeçkinJincai SuTurkay TurkogluJindrayani Nyoo PutroTurker DagdelenJing ChenTushar Kanti DasJing LiTyler J. GrimmJing YangUdhay SundarJingfa LiUgo LafontJingjing GaoUlderico SantamariaJinglei OuyangUmapada PalJingquan HanUmapathi ReddicherlaJingran BiUmmi Hani Binti AbdullahJingyun WuUrszula MizerskaJin-Kyun LeeUrtė BubnienėJinlong LiuÜsame Ali UscaJinxin GuoV. KavimaniJinxin LiV. L. MangeshJinyan WangVadde RamuJiri ChvojkaVadivel SethumathavanJiři HorskyVaidhegi KugarajahJiri MajznerVairavel ParimelazhaganJiujun ChengVaishakh NairJiunren HwangVakayil PraveenJizhen ZhangValentin NastasaJoachim F. R. Van GuyseValentina LoganinaJoachim StumpeValentina TomeiJoan KingValer MicleJoana FangueiroValeria LotitoJoanna KorzekwaValeryia KasnerykJoanna KurczewskaVan Chien PhamJoanna MarszałekVan Thanh NguyenJoanna PagaczVan Thanh Tien NguyenJoanna RaczkowskaVan Tu DuongJoão BassoVan-Thai TranJoão Carlos Mesquita CoelhoVarvara GribovaJoão Manuel Mendes CaramesVasileios VavourakisJoão PinaVasilica PopescuJoaquín Hernández-FernándezVedha Hari B. NarayananJochen VogtVeerababu PolisettiJohan BuitenhuisVelmurugan RamanJohn DetermanVengatesan Muthukumaraswamy RangarajJohn G. HardyVenkata MentaJohn K. JacksonVenkatesh ChelvamJohn KilbaneVera JencovaJohn LoveVera MazankovaJohn MisasiVera PiresJohn S. MitchellVera V. BudaevaJohn VlachopoulosVeronica MucciJohn W. HutchinsonVeronique MichaudJojo JosephVesna M. VučurovićJolanta KowalskaVesna RadojevićJolita OstrauskaiteViacheslav BalobanovJon Del ArcoViacheslav SlesarenkoJon Einar DahlVibhu PrasadJonas AlexandreVictor A. AlexeevJonathan L. CapeVíctor De La Asunción NadalJonathan Sabaté Del RíoVictor IzraylitJongho KimVíctor Javier Cruz-DelgadoJonghyun ChoiVictor NazarychevJong-Hyun KimVictor S. BalderramaJong-Min JeonVictoria Gonzalez-PedroJong-Seok ParkVijay MishraJoo-Hyung KimVijay TambrallimathJoohyung LeeVijayakumar SekarJoon Kyu LeeVikram DeyJoonwon BaeViktor Korzhikov-VlakhJordana GeorginViktor V. KlimovJordi FarjasViktor ZvarychJordi MartiViktoria RajtukovaJörg SchnaußViktoriya KonovalovaJorge PeixinhoVinam PuriJorge SilvaVinay SainiJorge Velásquez-CockVincenzo VillaniJosé A. MartinsVinh NguyenJosé Antonio PalenzuelaVinicius Tadeu SantanaJosé Bonilla-CruzVinod AyyappanJosé Francisco Serrano ClaumarchirantVinod KadamJose G. Munguia-LopezVinod Sathish KumarJosé Ginés Hernández-CifreVioletta GilJosé Javier Relinque MadroñalVioletta KytinouJose L. ConchaVipan KumarJosé Luis Rivera-ArmentaVipin AmoliJose Luis Sanchez-SalvadorVirender SinghJosé Maria LassoVishnu SaseendranJose Rodrigo Córdova AlarcónVišnja Gaurina-SrčekJose TahaVitaliy G. ShevchenkoJosé Vega- BaudritVito GiganteJosef JůzaVittorio LoddoJosé-Marie Lopez-CuestaViveca WallqvistJoseph AssaadVivek NarisettyJoseph DeitzelVivek SubramanianJoseph GovanVlad CarleucuJoseph J. MarcinkoVlad Constantin TofanJoseph W. LowdonVladimir A. YakushinJoshua E. S. J. ReidVladimir Alonso Escobar-BarriosJosip MiklečićVladimir G. SergeyevJosu AmorebietaVladimir KurbatovJuan C. MejutoVladimir M. Gun’koJuan CastilloVladimir PankratovJuan F. VegaVladimir ReukovJuan Hernández-CorderoVladimir V. BotvinJuan José Benvenuta-TapiaVladimir VolkovJuan José López-CelaVladislav SemakJuan Luis PrietoVlastimil BílekJuan Rodrigo Vélez-CorderoVolker SchöppnerJuana BenaventeVolodymyr KorolovychJuana María NavarroVu Anh DoanJudita GražulytėWael A. AltabeyJudith M. BragancaWagner TenfenJuiche LinWahid FerdousJuicheng ChangWajaht A. ShahJukka SeppäläWaldemar A. MarmisolléJulia CassaniWaldemar PerdochJulia LorenzoWaldemar PydaJulian Ajith ThambooWaldemar ŚwiderskiJulian ChojnowskiWaleed AhmedJun Cong GeWaqas AhmadJun LiuWarren BluntJun SawaiWaseem Sharaf SaeedJun SunWei CaoJun WangWei DuJun XieWei FeiJun YanWei LuJung-Hong MinWei ShaoJung-Hoon ParkWei WeiJung-Il SongWei WuJunhui JiWei YangJunichi FukudaWei ZhaoJunren WangWeidong HeJunyi SunWeihong WangJurgen LangeWeikang LiJurij ReščičWeiting LinJustyna DzięciołWenbin ZhouJustyna KobryńWenchang HuangJustyna SulejWenchao ZhaoJustyna ZygmuntowiczWendel SilvestreJutta RiegerWenhao LiJuyoung KimWenjia WangJyhherng ChenWenjuan ShiK. M. Faridul HasanWenlong ZhangKadarkaraisamy MariappanWentao LiuKadir BilisikWenten KuoKadiyala Chandra Babu NaiduWerner GoedelKailas K. MaliWerner PauerKaiyang YinWesley Lyeverton Correia RibeiroKajal GhosalWieslawa CiesinskaKajetan DabrowaWiktor BerskiKakan WongWilliam ArcherKalpana NagpalWilliam ClowerKamal Hany HusseinWilliam RassmanKamal I. AlyWilliam T. HellerKamal KansouWillian KenyonKamal SharmaWinifred ObandeKam-Chau WuWojciech JerzakKamila MizeraWojciech KujawskiKan DingWojciech WalachKanagaraj PalsamyWojciech ŻurowskiKang ZhaoWojnarovits LaszloKarim Alireza MohammadWolfdietrich MeyerKarim AlyWolfgang KernKarim TanjiWolfgang QuappKarin MolenveldWon-Min ParkKarla Čech BarabaszováWoo-Jin SongKarolina WiszumirskaWook-Bae KimKarthik KannanWorawan PanpipatKarthik KumaraWout De BackerKarthik Ram RamakrishnanWurong JianKartick Kumar SamantaXabier GómezKaruppasamy KaruppasamyXian GuijunKaruppusamy LoganathanXiang ChenKashif IshfaqXiangguo TengKashma SharmaXianglei ZhengKaspar M. B. JansenXiao HeKasza GyörgyXiaobo DongKatalin PereiXiaodi WangKatarina Dimic-MisicXiaogang ChenKatarína GajdošováXiaohong WangKatarina GmucovaXiaoju WangKatarzyna BuczkowskaXiaoliang QiKatarzyna JaszczXiaolin FanKatarzyna KrukiewiczXiaolong HaoKatarzyna LawinskaXiaomeng LiuKatarzyna Małolepsza-JarmołowskaXiaoqing MiaoKatarzyna PanasiukXiaoqing ZhaoKateryna FatyeyevaXiaoqun ZhuKatherine Lynn O'ShaughnessyXiaoting HeKathleen D. CusickXiaowei ChengKatona GáborXiaowei YuKavalipurapu Venkata TejaXiaoyi ChenKawee SrikulkitXiaozhou JiKazım KöseXiaozhou MaKazutaka HirakawaXi-Le HuKei NishidaXin TongKeith RochfortXinda LiKenan SongXinfang ZhangKen-ichi NomuraXing ZhouKening LangXinglei LiuKenji TakadaXinhua YaoKentaro YoshidaXiyuan ChenKesaven BhubalanXiyue ZhangKetan M. RanchXubo LuoKeum Hwan ParkXue LiuKevin WardXuehua WangKeyang WuXuejun ZhangKhadija JavedXuesong JiangKhadija MaqboolXuesong LiKhalid UmarXuhao ZhouKhalil AhmadXuman ChenKhalisanni KhalidXurui ZhangKhan NekoueeYacine OussarKhandoker SalemYan SuffrenKhang Wei Tan



